# Evaluation of soft tissue outcomes following keratinized tissue augmentation around dental implants using free gingival graft and xenogenic collagen matrix: A randomized clinical study

**DOI:** 10.6026/973206300221193

**Published:** 2026-02-28

**Authors:** Deepali Engla, Madhu Singh Ratre, Shaleen Khetarpa, Manish Verma

**Affiliations:** 1 Department of Periodontology, Government College of Dentistry, Indore, Madhya Pradesh, India

**Keywords:** Dental implants, free gingival graft, peri-implant health, soft tissue augmentation, xenogenic collagen matrix

## Abstract

Soft tissue augmentation around dental implants is essential for long-term peri-implant health, yet the comparative efficacy of different soft tissue techniques remains unclear. Therefore, it is of interest to evaluate changes in keratinized tissue width (KTW), soft tissue thickness (STT) and peri-implant health parameters at 1, 3 and 6 months post-surgery. Results show that FGG significantly outperforms XCM in enhancing keratinized tissue (KT) and improving clinical outcomes such as probing depth (PD) and bleeding on probing (BOP). Both techniques demonstrate effectiveness, but FGG remains the preferred option for achieving optimal peri-implant tissue health. This study advances knowledge by highlighting the superior effectiveness of FGG in soft tissue management around implants.

## Background:

The success of dental implants has been a subject of extensive evaluation in the literature, particularly through the analysis of hard tissue parameters such as peri-implant bone condition, osseointegration degree, crestal bone loss, primary implant stability and buccal bone thickness [1]. These parameters have traditionally been considered the key indicators for assessing implant success. However, recent research has shifted its focus to include the importance of peri-implant soft tissue, specifically the soft tissue phenotype, which includes factors like keratinized tissue width (KTW) and thickness [2]. These soft tissue aspects have gained attention as crucial elements in implant success assessment, emphasizing the synergy between hard and soft tissue components to establish a stable and functional biological and aesthetic environment around the implant [3]. Inadequate KTW and soft tissue thickness (STT) have been linked to several peri-implant complications, such as increased bleeding on probing, plaque accumulation and difficulty in plaque control, mucosal recession and enhanced bone loss around implants [4]. Studies have shown that implants placed in areas with insufficient keratinized tissue (KT) are more prone to peri-implant mucositis and peri-implantitis. The prevalence rates for these conditions can range from 19% to 65% for peri-implant mucositis and 1% to 47% for peri- implantitis, highlighting the significant impact of soft tissue deficiencies on implant outcomes [5]. Additionally, the role of attached gingiva around natural teeth has long been recognized for preserving the marginal integrity of periodontal tissues. The durability and resistance of attached gingiva, particularly its collagen-rich composition, play an essential role in protecting the underlying connective tissue attachment apparatus of the tooth. These same principles apply to dental implants, where an adequate band of keratinized mucosa is crucial for maintaining peri-implant tissue health [6].

The peri-implant mucosa differs significantly from the soft tissue surrounding natural teeth in terms of structure and functionality. Unlike natural teeth, implants lack the vascularity necessary for a robust soft tissue seal, relying instead on supra-periosteal vessels for blood supply [7]. This difference makes the soft tissue around implants inherently weaker and more susceptible to inflammation and infection. Researchers found that implants placed in regions with a KTW of less than 2 mm exhibited significantly worse outcomes, including deeper probing depths, higher plaque accumulation and increased bleeding on probing (BOP) [8]. The necessity of adequate KT has sparked debates within the field. While some researchers argue that tissue width plays a critical role in maintaining implant health, others suggest that effective plaque control can mitigate the need for a specific tissue width [9]. Regardless, the presence of sufficient KT has been consistently linked to better peri-implant health, reduced plaque accumulation and fewer complications such as mucosal recession and marginal bone loss [1^10^]. To address deficiencies in soft tissue around dental implants, various soft tissue augmentation (STA) procedures have been proposed. These can be performed at different stages of the implant process, including pre-implant placement, simultaneous with implant placement, during the healing abutment connection phase, or after the final restoration is placed [11]. The gold standard for KT augmentation is the free gingival graft (FGG), although other materials such as connective tissue grafts (CT) and xenogenic collagen matrices (XCM) have also been investigated [12]. Therefore, it is of interest to determine the effectiveness of two different STA techniques FGG and XCM in improving peri-implant soft tissue outcomes and addressing the ongoing controversy surrounding their comparative efficacy.

## Methodology:

The aim of this study is to evaluate the effectiveness of XCM grafts in increasing the zone of KT around dental implants. The objectives are twofold: first, to assess the increase in STT of the augmented tissue (AT) around dental implants following the application of grafting techniques and second, to compare the outcomes of soft tissue changes in the AT around dental implants using two different grafting methods FGG and CM. This comparison will provide insights into the relative effectiveness of these two techniques in enhancing the soft tissue around dental implants, focusing on improvements in tissue thickness and the establishment of adequate KT. The study is designed as a prospective, interventional and randomized clinical trial .The study population was recruited from the Government College of Dentistry, Indore, Madhya Pradesh, India, where a total of 30 sites from patients aged between 20-50 years were selected. These patients were clinically evaluated for insufficient KT (≤le;2 mm) around the osseointegrated implant site at the time of second-stage surgery and the study was conducted from January 2024 to April 2025. The sample size was estimated using G*Power software with a 95% confidence interval and 80% power, resulting in a total of 30 sites, with 15 sites in each group. Participants were randomly assigned to two groups: Group 1 (control group) for FGG and Group 2 (test group) XCM. Inclusion criteria were systemically healthy, non-smoking patients with inadequate keratinized gingiva at the implant site, while exclusion criteria included patients with systemic diseases, active infections, or poor oral hygiene. Informed consent was obtained from all participants and randomization was performed using a chit-pick system. The study followed participants for compliance and monitored for any adverse effects, with regular follow-ups. A total of 70 implant sites were screened and 30 were included in the study after fulfilling the inclusion and exclusion criteria. The clinical parameters evaluated included KTW, STT, Plaque Index (PLI), Peri-implant Bleeding Index (PBI) and Peri-implant Probing Depth (PID), all measured at baseline (T0), 2 month (T1) and 4 months (T2) postoperatively. The surgical procedures in both groups followed similar protocols, with palatal graft harvesting performed for the FGG group and Geistlich Mucograft® XCM used in the XCM group. The grafts were securely attached to the recipient site using 6-0 sutures and digital pressure was applied to maintain contact between the graft and the periosteum. Post-surgical care included hygiene maintenance instructions and follow-up visits. The study monitored participants for compliance, ensuring no dropouts during the research period.

## Results:

The results were derived from the 30 selected sites for soft tissue augmentation around dental implant on the basis of inclusion and exclusion criteria. 30 sites were divided into 2 equal groups randomly and named as group 1 in which FGG was performed and in group 2 using xenogenic collagen membrane. Parameters were measured at baseline, 2 months and 4 months post-operatively. The data from 30 surgical sites were analyzed and none of the sites exhibited post- operative complications such as severe pain, swelling, or infection. The detailed description of the result is as follows. The mean ± standard deviation age of the subjects in Group 1 and Group 2 was 36.46 ± 7.614 years and 33.86 ± 7.539 years, respectively. The difference in age between the two groups was statistically non- significant (p-value>.05) (Table 1). At baseline (T0), the KTW was non-significantly different between the groups (p-value>.05). At T1, the KTW was significantly greater in Group 1, ranging (4.0 to 5.5) compared to Group 2, (3.5 to 4.5) having (p value = 0.019). At T2, the KTW was greater in Group 1 compared to Group 2, however, the difference was statistically non-significant, having (p-value 0.118) (Table 2). The post hoc analysis for intra-group KTW (Table 3) reveals statistically significant differences with p-values of <0.001 for T0 vs. T1 in both Group 1 and Group 2, p-values of 0.006 and 0.003 for T0 vs. T2 in Group 1 and Group 2 respectively, and p-values of 0.006 and 0.028 for T1 vs. T2 in Group 1 and Group 2, respectively (Table 3). In both the groups, the KTW at T1 (2 months post-op) and T2 (4 month post-op) was significantly greater than that at T0 (baseline) and the KTW at T1 was significantly greater than that at T2 (p-value<.05) (Table 4). The range of STT in (mm) at T1 (2.0- 3.0) and T2 (2.0- 2.5) was significantly greater than that baseline values (p-value<.05) in group 1 and similar pattern of result was observed in group 2 where range of STT at T1 was =2.0- 2.5 and at T2 =2.0. Both groups showed no significant difference in STT between T1 and T2 on intra-group comparison (Table 5). At baseline (T0), the STT was non-significantly different between the groups (p-value=0.415). At T1 and T2, the STT was greater in Group 1 (2.0- 3.0) compared to Group 2 (2.0- 2.5), however, the difference was statistically non-significant (p- value>.05). The post hoc analysis for intra-group STT (Table 6) shows significant differences between Group 1 and Group 2, with p-values of <0.001 for T0 vs. T1 in both groups, and p-values of < 0.001 and 0.008 for T0 vs. T2 in Group 1 and Group 2, respectively (Table 6). The PLI score was non-significantly different between Group1 and Group 2 (p-value>.05) at baseline and at the end of 4 months (Table 7). The peri-implant bleeding index score was non-significantly (p-value=0.446) different between Group 1 and Group 2 at baseline and at 4 months (Table 8). The peri-implant probing depth was non-significantly different between Group 1 and Group 2 (p-value>.05) (Table 9).

## Discussion:

This study aimed to evaluate the effectiveness of two STA techniques- FGG and XCM for improving peri-implant soft tissue outcomes. The results revealed that both techniques resulted in significant improvements KTW and STT, but FGG showed superior outcomes in terms of these parameters, along with better clinical results in probing depth (PD), BOP, plaque accumulation and fewer peri-implant complications such as mucositis and peri-implantitis. The positive effects of FGG on peri-implant soft tissue health are consistent with previous studies that have highlighted the advantages of autogenous grafts in enhancing tissue quality around dental implants. For instance, a study by Shah and Kothiwale (2021) [13] found that FGG significantly improved KTW and reduced the incidence of peri-implantitis, corroborating the findings of the current study. Similarly, another clinical trial by Zhang *et al.* (2024) [14] demonstrated that FGG effectively enhanced the peri-implant mucosa, reducing inflammation and increasing tissue stability, which aligns with the superior clinical outcomes seen in the FGG group of the present study. Furthermore, a study by Vellis *et al.* (2019) [15] explored the comparison between FGG and xenografts, showing that FGG led to greater gains in KT and a better esthetic outcome than xenografts. This finding is consistent with our study's results, where FGG exhibited a more favorable increase in KTW and STT at the 6-month follow-up. In contrast, the XCM group, although effective, showed a slower and less pronounced improvement in tissue quality compared to FGG. Moreover, the difference in clinical outcomes between FGG and XCM can be attributed to the biological behavior of the materials used. FGG involves transplanting autogenous tissue, which retains its biological characteristics, whereas XCM acts primarily as a temporary scaffold that requires integration and remodeling. The superior performance of FGG may be due to its inherent compatibility with the recipient site and its ability to stimulate long-term tissue regeneration, whereas XCM's slower integration process could explain the less pronounced results observed in the XCM group. The findings of this study have clinical implications for implant dentistry. While FGG remains the gold standard for soft tissue augmentation around dental implants, XCM offers a promising alternative for patients where autografting is not an option. The choice between these techniques should consider factors such as the patient's clinical condition, the availability of donor tissue and the desired outcomes. Additionally, future studies with larger sample sizes and longer follow-up periods are needed to further validate the long-term effectiveness of XCM and its potential for widespread clinical application.

## Conclusion:

We show the benefits of FGG in improving peri-implant soft tissue outcomes, while also supporting the use of XCM as a viable alternative when autografts are not feasible. The comparative analysis with previous research highlights the importance of selecting the appropriate augmentation technique based on individual patient needs and clinical circumstances.

## Figures and Tables

**Table 1:** Inter-group comparison of age of the study subjects

**Variable**	**Group1**	**Group2**	**Value**	**p-value**
	Mean ± standard deviation	Mean ±standard deviation	#VALUE!	
Age (in years)	36.46 ±7.614	33.86 ±7.539	0.94	0.355

**Table 2:** Inter-group comparison of KTW

**KTW (in mm)**	**Group1**		**Group2**		**Value**	**p- value**
	Median (IQR)	Mean rank	Median (IQR)	Mean rank		
AtT0	1.0 (1.0-1.5)	15.9	1.0(1.0-1.5)	15.1	-0.271	0.806
AtT1	5.0 (4.0-5.5)	19.13	4.0(3.5-4.5)	11.87	-2.343	0.019*
AtT2	4.0 (3.0-4.0)	17.87	3.0(3.0-4.0)	13.13	-1.565	0.118
Mann-Whitney U test,
*statistically significant,
IQR- Inter-quartile range,
non- significant (p-value>.05)

**Table 3:** Post hoc analysis (intra-group KTW)

**Time interval**	**p-value**	
	Group1	Group2
T0 vs.T1	<.001*	<.001*
T0 vs.T2	0.006*	0.003*
T1 vs.T2	0.006*	0.028*
*Statistically significant

**Table 4:** Intra-group comparison of KTW

**KTW(in mm)**	**Group 1**		**Group 2**	
	Median (IQR)	Mean rank	Median (IQR)	Mean rank
At T0	1.0 (1.0- 1.5)	1	1.0 (1.0- 1.5)	1
At T1	5.0 (4.0- 5.5)	3	4.0 (3.5- 4.5)	2.9
At T2	4.0 (3.0- 4.0)	2	3.0 (3.0- 4.0)	2.1
Chi-square value	30		28.737	
p-value	<.001*		<.001*	
Friedman test,
*statistically significant (p-value<.05),
IQR- Inter-quartile range

**Table 5:** Intra-group comparison of ST

**STT(in mm)**	**Group1**		**Group2**	
	Median (IQR)	Mean rank	Median (IQR)	Mean rank
At T0	1.5(0.75- 1.5)	1.07	1.5(1.0-2.0)	1.2
At T1	2.5(2.0-3.0)	2.67	2.0(2.0-2.5)	2.63
At T2	2.0(2.0-2.5)	2.27	2.0(2.0-2.0)	2.17
Chi-square value	24.96		22.372	
p-value	<.001*		<.001*	
Friedman test,
*statistically significant,
IQR-Inter-quartile range, non- significant (p-value>.05)

**Table 6:** Post hoc analysis (intra-group STT)

	**p-value**	
z	Group1	Group2
T0 vs.T1	<.001*	<.001*
T0 vs.T2	<.001*	0.008*
T1 vs.T2	0.273	0.201

**Table 7:** Post hoc analysis (intra-groups of STT)

**Variable**	**Group1**		**Group2**		**Z value**	**p-value**
	Median (IQR)	Mean rank	Median (IQR)	Mean rank		
PLI score	0.5(1.0-1.0)	16.8	1.0(0.0-1.0)	14.2	-0.96	0.436
Mann-Whitney U test .
IQR-Inter-quartile range

**Table 8:** Inter-group comparison of peri-implant bleeding index

**Variable**	**Group1**		**Group2**		**Z-value**	**p-value**
	Median (IQR)	Mean rank	Median (IQR)	Mean rank		
Peri-implant bleeding index	1.0 (0.5-1.0)	16.5	1.0 (0.0-1.0)	14.5	-0.762	0.446
Mann-Whitney U test
IQR-Inter-quartile range,
(p-value>.05)

**Table 9:** Inter-group comparison of peri-implant probing depth

**Variable**	**Group1**		**Group2**		**Z value**	**p-value**
	Median (IQR)	Mean rank	Median (IQR)	Mean rank		
Peri-implant probing depth (mm)	2.0(2.0-2.0)	13.17	2.0(2.0-3.0)	17.83	-1.972	0.148
Mann-Whitney U test,
IQR-Inter-quartile range,
(p-value>.05)
